# Engineering *Escherichia coli* K12 MG1655 to use starch

**DOI:** 10.1186/1475-2859-13-74

**Published:** 2014-05-21

**Authors:** Luis Manuel Rosales-Colunga, Agustino Martínez-Antonio

**Affiliations:** 1 Departamento de Ingeniería Genética, CINVESTAV, Km 9.6 Libramiento Norte Carr. Irapuato-León, 36821 Irapuato, Gto, México; 2 Current address: Facultad de Ingeniería, Universidad Autónoma de San Luis Potosí, Av. Dr.Manuel Nava 8, Zona Universitaria, San Luis Potosí, SLP, México

**Keywords:** Amylase, Synthetic biology, Adaptive strain, Maltodextrins, Bacteria, *E. coli*

## Abstract

**Background:**

To attain a sustainable bioeconomy, fuel, or valuable product, production must use biomass as substrate. Starch is one of the most abundant biomass resources and is present as waste or as a food and agroindustry by-product. Unfortunately, *Escherichia coli*, one of the most widely used microorganisms in biotechnological processes, cannot use starch as a carbon source.

**Results:**

We engineered an *E. coli* strain capable of using starch as a substrate. The genetic design employed the native capability of the bacterium to use maltodextrins as a carbon source plus expression and secretion of its endogenous α-amylase, AmyA, in an adapted background. Biomass production improved using 35% dissolved oxygen and pH 7.2 in a controlled bioreactor.

**Conclusion:**

The engineered *E. coli* strain can use starch from the milieu and open the possibility of optimize the process to use agroindustrial wastes to produce biofuels and other valuable chemicals.

## Background

The development of biomass-based processes is key to the establishment of sustainable and petroleum-independent industry [1]. Therefore, the production of valuable products, such as biofuels, lactic acid, and ferulic acid, is currently based on the use of biomass to close the CO_2_ cycle. In this cycle, the CO_2_ is integrated into biomass, released, and reincorporated again into biomass, which differs from the use of fossil counterparts.

Biomass resources range from lignocellulosic sources to grains that are usually employed for human consumption. Despite the great amount of biomass available in crops, their use as human food raises concern for their role in biotechnological processes. Thus, discovering alternatives sources is critical. Many of the processes under development focus on hydrolysis of lignocellulosic materials for microbial-biomass production. However, there are many other sources of biomass available, including agroindustrial by-products or wastes such as cheese whey [2,3], olive mill waste-water [4], cassava bagasse, citrus-processing residues [5], and manure [6]. From these biomass sources, there is the potential to obtain valuable products.

One of the main biomass components is starch [7]. The predominant natural sources of starch are maize, potato, wheat, rice [8], and other diverse tubercles that are widely used in food-processing industries and generate a large amount of waste. For instance, potato pulp, which is a by-product generated from potato processing, usually contains a considerable amount of starch and fibers, which could represent an excellent source for the production of valuable products, such as lactic acid [9]. Furthermore, in plants and particularly tubercles, almost every carbon storage tissue has elevated starch content. These organic materials are largely neglected as potential starch sources.

To take advantage of starch-rich bio-resources, it is necessary to develop microbes capable of hydrolyzing starch and utilizing their products. To achieve this goal, utilization and adaptation of hydrolytic enzymes is necessary. In industry, hydrolysis of these biomass materials requires enzymatic and/or acid hydrolysis pre-treatment prior to fermentation. A bacterial strain that develops both hydrolysis and fermentation would be extremely valuable.

The bacterium *E. coli* is widely used as a model organism both in research and industrial processes, and its genome encodes a cytosolic α-amylase, AmyA. It is of great interest to secrete this enzyme in the milieu to determine its exogenous hydrolytic capacity. Among secretion signal peptides, the FhuD signal shows high secretory activity. This activity could be due to its capacity to use the two secretory pathway systems present in *E. coli*: Tat and Sec [10].

For starch hydrolysis, α-amylase should be expressed by the bacterium at basal levels to immediately begin starch hydrolysis in the milieu. Ideally, the resultant products of starch degradation, maltose and maltodextrins, should further activate AmyA expression. In *E. coli*, expression of native genes related to maltose/maltodextrin catabolism and their transport is controlled by 5 operons that include 10 genes. These consist of ABC transporters and catabolic enzymes regulated by the transcription factor MalT [11-13]. However, there is no evidence that the *amyA* native gene is part of this system, and it is not regulated by MalT. Genes regulated by MalT show basal expression levels when maltose/maltodextrins are not present [11]. This expression is augmented in the presence of maltose/maltodextrins that function as co-activators of MalT, and maltodextrins of up to seven units can be transported into the cytosol to sustain *E. coli* growth [14].

Strain development to increase the efficiency of microorganisms has been an important objective of the biotechnology industry for decades [15]. Among existing methods that improve strain performance, the selection of spontaneous adaptive mutants is a simple, and in most of cases, effective technique. Based on reports of catabolic adaptive mutants, the most common mechanisms described are conversion of an induced system to a constitutive or de-repressed system [16,17], and increasing permeability and substrate uptake [16,18].

In this study, we selected adaptive mutants to grow on starch-containing media and use a synthetic biology approach based on BioBricks technology, which employs restriction-site standardized genetic parts, to develop an *E. coli* strain able to consume starch. Our aim was to engineer an *E. coli* strain that can serve as a starting point to be used as microbial platform to use agroindustrial wastes to produce biofuels and other valuable compounds.

## Results and discussion

### Adapted *E. coli* strain selection

**Figure 1:**
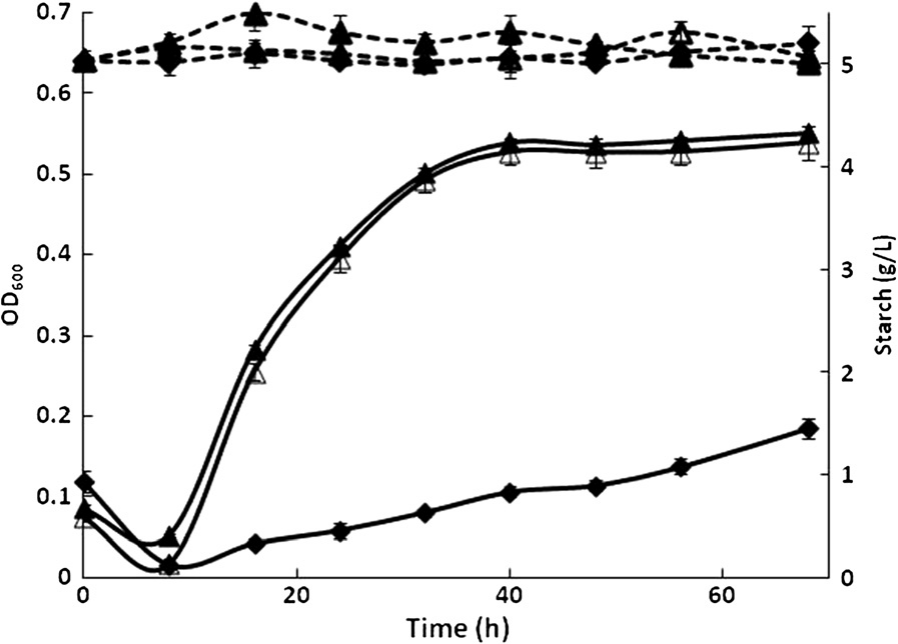
**Growth kinetics of adaptive strains on M9 plus starch media.** Growth profiles are shown as continuous lines whereas starch content in supernatants are shown as discontinuous lines. WT (♦), WTa1 (∆), and WTa6 (▲) strains.

We noted growth of wild-type *E. coli* (WT) after a long cultivation time (6–8 days) in some liquid cultures with starch as the carbon source (data not shown). For this reason, a sample of the WT strain was cultured on solid M9 plus starch plates to identify and select colonies capable of faster growth on starch. After 120 h at 37°C, colonies were observed on one of the plates. An average of 23 colonies per plate were observed after incubation for 144 h. Each of these colonies was replicated on fresh plates, and most grew after 48 to 96 h at 37°C. However, six of these adapted strains grew faster and were thus selected for further growth evaluation in liquid M9/starch (5 g/L). Most of these selected strains showed slow but continuous growth. From these, the strains identified as WTa1 and WTa6 grew fastest and yielded the highest biomass. The growth kinetics of WT compared to the adapted strains WTa1 and WTa6 are shown in Figure 1. Slight growth was observed in the WT strain, whereas the adapted strains reached an optical density (OD_600_) of 0.5 after 40 h.

Shibuya *et al.*[19] reported similar growth profiles for other adapted strains as well as extracellular amylolytic activity from the adapted strains. However, in the present work, the adapted strains did not show amylolytic activity because no starch degradation was observed (Figure 1). Thus, the observed growth could be due to better use of small maltodextrins or additional contents in the commercial starch employed, the presence of which cannot be detected by the method used here. Indeed, the initial culture media was analyzed by high-performance liquid chromatography (HPLC). The results indicated a mix of maltodextrins of 3–5 glucose units and trace amounts of glucose, fructose, maltose, and sucrose, in addition to the starch content. Because similar biomass yields were obtained from the two adapted strains, we used the WTa6 strain in all subsequent experiments.

**Figure 2:**
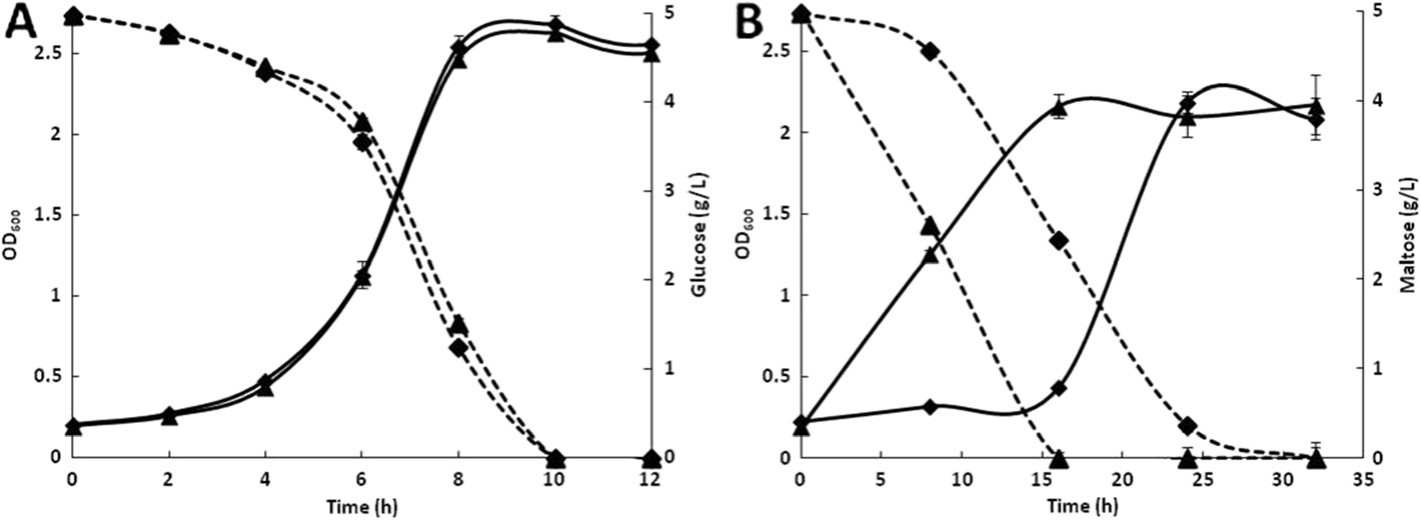
**Growth kinetics of WT and a starch-adapted strain.** Growth profiles (continuous lines) and sugar consumption (discontinuous lines) of the WT (♦) and WTa6 (▲) strains using glucose **(A)** and maltose **(B)**. Error bars are not always visible because they are smaller than the symbol.

Considering that, in adaptive mutants, the most commonly described adaptive mechanism involves the de-repressed system, we evaluated the use of maltose as a substrate by the WTa6 strain. The growth kinetics and sugar consumption of the WT and WTa6 strains using glucose (A) and maltose (B) are shown in Figure 2. The growth and glucose consumption kinetics of the WT and WTa6 strains were very similar (Figure 2A). However, the WTa6 strain immediately metabolized maltose when this sugar was used as a substrate, and growth began immediately. In contrast, the WT strain did not begin to metabolize maltose until after 8 h, and the growth lag phase was almost 16 h. This observation could explain the better performance of WTa6 using starch because this strain has improved maltose/maltodextrins metabolism. It appears that this system is constitutively expressed and/or maltodextrin permeability is improved in this strain. Similar results have been previously reported. Dardonville and Rainbaud [20] isolated a mutant strain with constitutive expression of the *mal* regulon, and some of these mutants contain mutations in the *malT* gene.

### Design and expression of α-amylase

Because the adapted strains did not show amylolytic activities, it is possible that the expression of exogenous α-amylase might improve starch utilization by the adapted strain and further increase the yield of biomass or any desired product derived from starch. The *E. coli* genome encodes the cytosolic α-amylase, AmyA. The reported substrates for this enzyme are maltodextrins with least six glucose units, amylose, and starch. Amylopectin is used less effectively [21].

To increase the uptake of carbon sources derived from starch, we engineered secretion of the AmyA enzyme using the FhuD signal peptide. By analyzing published microarray studies, we found that among the genes that are regulated by MalT, *malE* shows the highest response [22,23]. Therefore, we selected the *malE* promoter region to be used in our synthetic genetic construction. In this way, α-amylase expression and secretion could be coupled to the maltose/maltodextrin system. The plasmid pAM encodes the *amyA* construct in the plasmid pSB1A2 (see Methods section).

**Figure 3:**
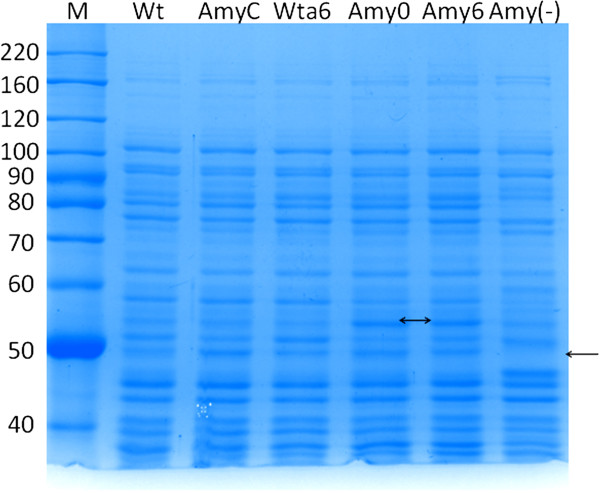
**Analyses using 10% SDS-PAGE from cultures with 5 g/L maltose.** M indicates molecular mass marker (kDa).

Next, the WT and WTa6 strains were transformed with the pAM vector to obtain the Amy0 and Amy6 strains, respectively. To assess the performance of pAM, WT, AmyC (WT with pSB1A2 as a control), WTa6, Amy6, Amy0, and Amy(−) (*amyA* gene deleted) strains were cultivated using maltose as a carbon source. The protein expression profiles of these strains are shown in Figure 3. The expression profiles were similar in all strains, except for an extra band observed between 50 and 60 kDa in the Amy6 and Amy0 strains (double arrow). Because the molecular weight of native α-amylase is approximately 56 kDa and the estimated molecular weight of the modified amylase is approximately 59 kDa, it is reasonable to propose that the extra band corresponds to the AmyA protein plus the FhuD signal peptide. Moreover, this band is absent in the Amy(−) strain (single arrow). These results suggest that the engineered α-amylase is expressed in the Amy strains.

### α-amylase secretion and bacterial growth

**Figure 4:**
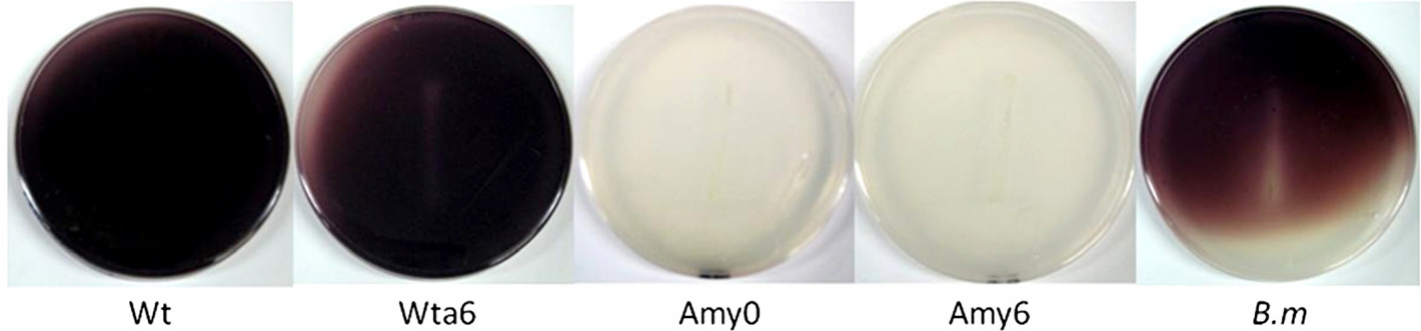
**Qualitative secreted amylase activity.** Lugol-staining of M9 plus starch plates where WT, WTa6, Amy0, Amy6, and *Bacillus megaterium* were cultured. Stained plates indicate no starch degradation. Clear plates indicate starch degradation by the amylase.

To hydrolyze starch in the milieu and use its hydrolysis products for growth and/or metabolite synthesis, α-amylase must be expressed and secreted by *E. coli*. The *E. coli* strains WT, WTa6, Amy0, Amy6, and *Bacillus megaterium* (used as a positive control) were cultured on M9-agar plates containing starch. As expected, slight growth was observed with the WT strain, whereas the adapted strains WTa6, Amy6, and *Bacillus megaterium* showed considerable growth. Expression of *amyA* in a WT background (Amy0) caused significant growth with starch as the only carbon source. The growth pattern was similar to that observed in the Amy6 and WTa6 strains. Additionally, the plates were stained with lugol solution, which stains starch as dark blue, to determine the amount of residual starch. Thus, starch degradation (clear plates) can be correlated with the extracellular activity of the expressed amylase. The qualitative results are shown in Figure 4. The plates on which the strains harboring the pAM plasmid were cultured were completely clear, indicating that the starch had been fully degraded. In fact, these plates were clearer than the plate with *Bacillus megaterium* growth, a bacterium that is known to secrete amylases into the media [24]. In contrast, plates growing strains without pAM did not present clear zones after staining with lugol. Therefore, we conclude that the amylase encoded in the pAM is functional and secreted to the milieu, where it is active.

### Improved biomass yield from starch

**Figure 5:**
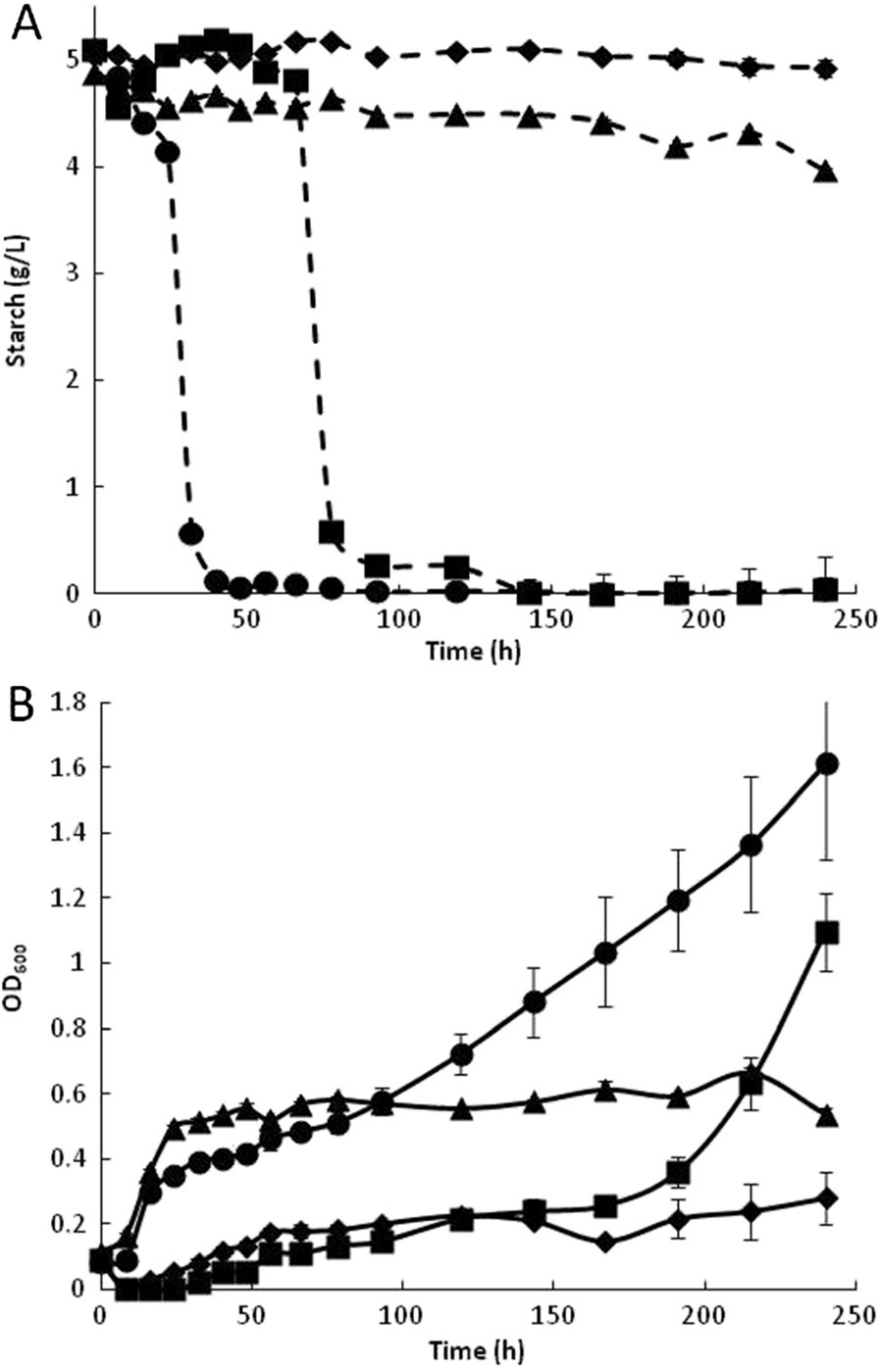
**Starch degradation and growth kinetics of strains grown on starch.** Kinetics of starch degradation **(A)** and growth **(B)** of the following strains: WT (♦), WTa6 (▲), Amy0 (■), and Amy6 (●). Error bars are not always visible because they are smaller than the symbol.

As discussed, adapted strains can grow in minimal media with starch as the sole carbon source despite any detectable amylolytic activity. Moreover, the genetic construct encoded in the plasmid pAM permits secretion of a functional amylase. To investigate whether AmyA secretion improves the biomass yield, WT, adapted WTa6, Amy0, and Amy6 strains were cultured in liquid media using starch as the substrate. Starch degradation (A) and growth kinetics (B) of these strains are shown in Figure 5. The WTa6 strain began to grow after 8 h, reached an OD_600_ of 0.55 after 48 h of culture, and stopped growing. After 143 h, slight growth was observed and a maximum OD_600_ of 0.633 was achieved at 215 h. In these cultures, only a small decrease in starch concentration was observed. Additionally, the maltodextrin content in the final culture media of this strain decreased. This result indicates that the growth of this strain is due to better use of maltodextrins. The Amy0 strain started to hydrolyze starch after 40 h, with a minor decrease observed between 40 h and 66 h. Subsequently, strong use of starch was observed. Although amylolytic activity was observed and short maltodextrins were likely available, a slight increase in OD_600_ was observed before 167 h. This period was followed by strong growth that permitted the cultures to reach an OD_600_ of 1 unit. AmyA expression and secretion in the adapted background allowed the Amy6 strain to degrade starch from the start of the culture time. Therefore, starch was completely hydrolyzed in the first 50 h. This strain grew slower than the parental WTa6 in the first hours of culture; however, after 16 h, continuous growth was observed and a maximum OD_600_ of 1.6 was reached. The WT strain did not show starch degradation and only slight growth was observed.

**Figure 6:**
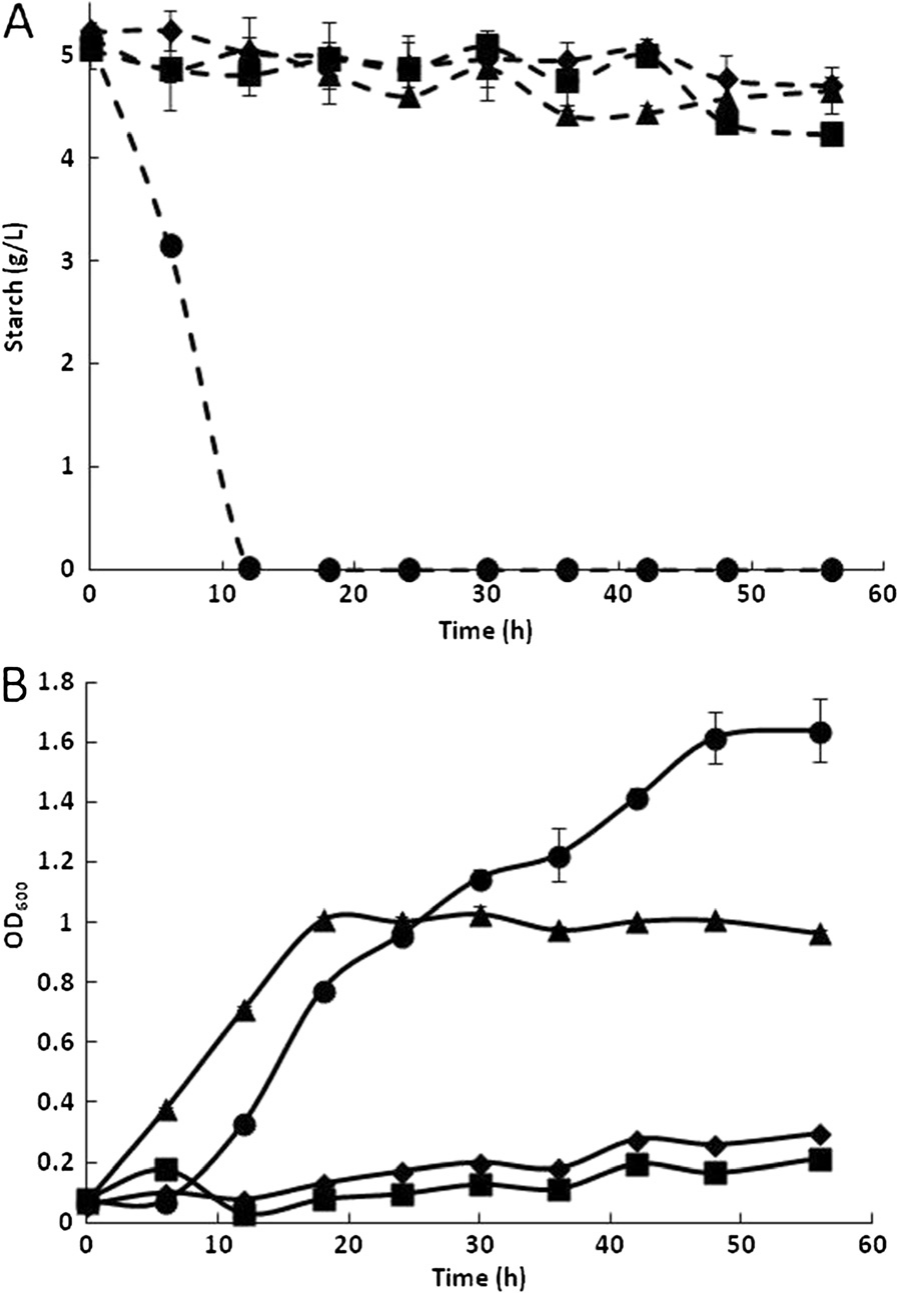
**Starch degradation and growth kinetics of strains previously cultured in starch.** Starch consumption **(A)** and growth kinetics **(B)** of the following cultures: WT (♦), WTa6 (▲), Amy0 (■), and Amy6 (●). Error bars are not always visible because they are smaller than the symbol.

**Figure 7:**
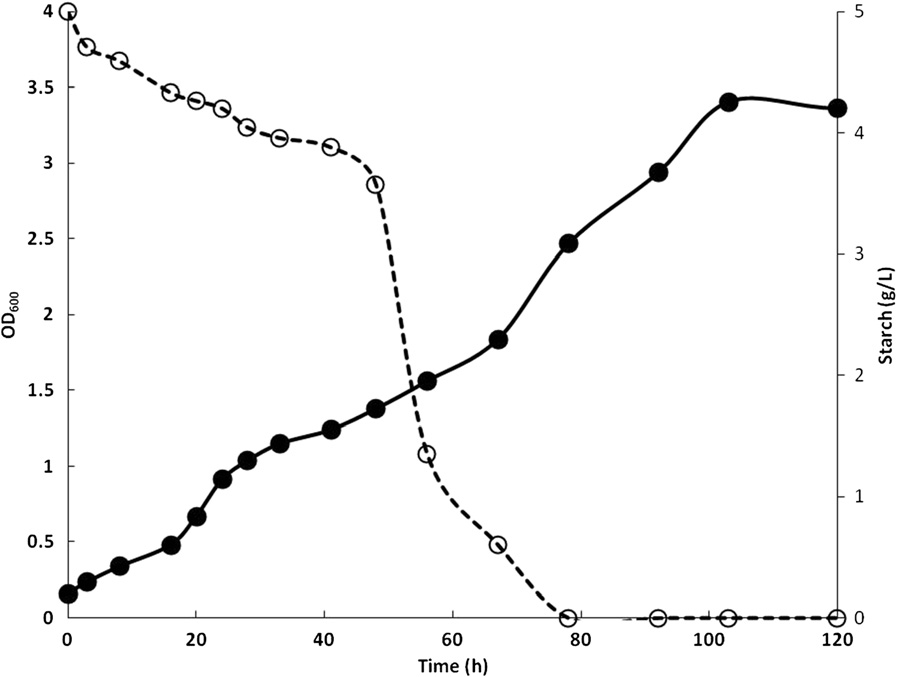
**Growth profile of the adapted strain with engineered amylase in a controlled bioreactor.** Starch degradation (○) and growth kinetics (●) of Amy6 in the controlled conditions of pH 7.2 and DO 35%.

According to the genetic circuit design, strains with the engineered AmyA need to metabolize a small fraction of starch to induce amylase production and secretion. Therefore, strong degradation of starch is observed only after a lag period (Figure 5). The metabolic advantage of the adapted strain led to earlier amylase production and utilization of the hydrolysis products (Amy6). The time to produce and secrete AmyA can be long, even when the pre-inoculants for these assays were grown using maltose (to pre-induce the maltose/maltodextrin system and *amyA* expression in the pAM construction). Although only maltotriose activates MalT, maltose and maltodextrins (maltotriose-maltoheptaose) can induce the system; however, maltose is a less effective inducer [13]. Based on these observations, the effect of pre-growth using starch was evaluated, and the results are shown in Figure 6. The time course of this experiment was shorter than that of previous experiments. The WT strain showed similar null growth as in the previous assays. Previous contact with starch did not improve the growth of the Amy0 strain or its degradation of starch until 56 h (Figure 6). However, the growth kinetics of WTa6 and Amy6 were improved. The WTa6 strain began to grow at the same time the culture was started, without a lag phase, and reached an OD_600_ of 1 after 56 h. This is 37% more biomass compared to the results shown in Figure 5. The Amy6 strain appears to hydrolyze starch immediately, with no residual starch content detected after 12 h (in comparison, with maltose in the pre-inoculant, it takes approximately 50 h). After a short lag phase of 6 h, this strain began to grow in a sustained manner and reached the highest growth of 1.64 OD_600_ units after 56 h. This is four times shorter than the 240 h this strain took to reach a similar OD_600_ (Figure 5) with maltose in the pre-inoculant. According to these results, the presence of maltotriose in the starch used in the pre-inoculant decreased the lag phase and the cultivation time. Moreover, under these conditions, the biomass yield of Amy6 was 60% higher than that of WTa6, whereas Amy0 and the WT only showed a slight growth. The Amy6 strain showed the best performance using starch. Therefore, we used a controlled bioreactor to evaluate the growth of this strain. Figure 7 shows the growth kinetics and starch degradation while maintaining the pH at 7.2 and the dissolved oxygen (DO) at 35%. Under these controlled conditions, growth was similar to the uncontrolled assay (Figure 6) during the first 56 h. However, upon maintaining the pH and DO, this strain continued growing until it reached an OD_600_ of 3.4 in 103 h (twice the OD_600_ in the previous assay). Under these conditions, starch was slowly hydrolyzed in the first 41 h and consumed until it was undetectable at 80 h. Furthermore, HPLC analyses of the final culture media showed that this strain consumed all carbohydrates.

These data highlight the potential of this strain to use starch to yield valuable products.

## Conclusions

In this study, we developed an *E. coli* strain capable of using extracellular starch as the sole carbon source. This was achieved by following a synthetic biology framework. The process takes advantage of the native capability of *E. coli* to use maltodextrins as carbon sources and a cytoplasmic amylase that was engineered to alter its transcription and secretion. The genetic construct generated a functional amylase with a signal peptide that permitted its secretion to the milieu. α-amylase expression and secretion allowed the bacterium to grow actively using starch as the sole carbon source. The starch degradation products activated α-amylase gene transcription, constituting a positive feedback circuit for amylase expression. Adapted strains grown on starch plates could be identified and selected by their ability to form colonies faster than WT. The use of these adapted strains as a background to harbor the engineered cytoplasmic amylase improved biomass yield using starch as substrate. Under controlled conditions (pH 7.2 and 35% DO), the biomass production of Amy6 was further increased. This work opens the potential to optimize the process to finally use this strain as a platform to produce added value products using starch as substrate.

## Methods

### Strains, culture media, and growth conditions

The strains and plasmids used in this work are shown in Table 1. *E. coli* K12 MG1655 was used to generate the strains designed for starch consumption. Amy6 and Amy0 contain the plasmid pAM (see genetic construction) to use starch as substrate.

**Table 1:** Strains and plasmids

**Strain/plasmid**	**Genotype or description**	**Reference**
Bm	*Bacillus megaterium*	[24]
WT	*Escherichia coli* K-12 MG1655	[25]
WTa1-6	WT, starch-adapted strains	This work
AmyC	WT with the backbone plasmid pSB1A2	This work
Amy0	WT with pAM	This work
Amy6	WTa6 with pAM	This work
Amy(−)	WT with *amyA* deleted	[26]
pSB1A2	BioBricks backbone plasmid	[27]
pSB1AK3	BioBricks backbone plasmid	[28]
pAM	pSB1A2 with engineered amyA	This work

Strains were grown routinely on LB plates. Pre-inoculants were grown in LB overnight, centrifuged at 10,000 rpm, and resuspended in M9 minimal media without a carbon source. They were inoculated in 125 mL or 250 mL baffled flasks with M9 plus carbon sources (5 g/L). Pre-inoculants for biomass yield from starch were obtained using M9 with 5 g/L maltose (Sigma) or soluble starch (Bio Basic Canada Inc.). Samples were taken periodically to evaluate growth at OD_600_, as well as for starch or sugar determination. Batch cultures were performed in a 3-L bioreactor (Applikon, The Netherlands) with a working volume of 1 L. The pH and dissolved oxygen (DO) were monitored online using autoclavable electrodes (AppliSense, The Netherlands). These parameters were automatically controlled using PID control with the My control^TM^ controller (Applikon) using 2.5 N NaOH and HCl solutions for the pH control and air for DO. The set points were 7.2 and 35% for pH and DO, respectively. Cultures were maintained at 37°C and stirred at 200 rpm with two six-blade Rushton turbines. BioXpert software (Applikon) was used for data acquisition.

Plate experiments were performed in M9 plates with 5 g/L of soluble starch. All assays were performed at 37°C. Carbenicillin (Sigma) at a final concentration of 100 mg/L was used in all cultures harboring the pAM plasmid.

### Selection of adapted strains

Two hundred microliters WT *E. coli* grown overnight were spread on M9/ starch plates and incubated at 37°C until colonies appeared. The resulting colonies were cultured again on fresh M9/ starch plates and incubated at 37°C to observe their growth. The strains with the fastest growth (i.e., colony formation) were further tested in M9/starch liquid media. A colony sample of each of the selected strains was taken and inoculated in 10 mL of M9/starch. The OD_600_ was monitored to select the fastest growing strains in liquid media.

### Genetic construction for starch hydrolysis

Primers were designed to amplify the genetic components used to hydrolyze starch (Table 2). These primers were designed to include the standardized prefix and suffix used in the BioBricks approach [29]. The prefix is comprised of *EcoRI* and *XbaI* restriction enzymes recognition sites, and the suffix contains *SpeI* and *PstI* recognition sites. These primer pairs and high fidelity PCR super mix (Invitrogen) were used to amplify the following DNA regions: i) the promoter region of *malE* (*malEp*) with prF and prR primers, ii) the signal peptide of *fhuD* (*fhuDsp*) with spF and spR primers, and iii) the α-amylase *amyA* (*amyA*) with amF and amR primers. The ATG codon of *malE* was changed to AGG (CTT in the primers), the suffix of *fhuD* and the *amyA* prefix were designed as suggested for the correct fusion of peptides [30], and with extra bases to be eliminated after PCR. The suffixes include two translation stop codons.

**Table 2:** BioBricks and primers used to obtain a genetic construct for starch hydrolysis

**Name**	**Description or Sequence (5'-3')**	**Reference**
BBa_B0030	Strong RBS	[31]
BBa_B1002	Artificial double-transcriptional terminator	[32]
*fhuDsp*	Signal peptide from *fhuD,* obtained by PCR with the primers spF and spR	This work
*malEp*	Promoter from *malE,* obtained by PCR with the primers prF and prR	This work
*amyA*	*amyA* gene*,* obtained by PCR with the primers amF and amR	This work
spF	GTTTCTTC**GAATTC**GCGGCCGCT ***TCTAGA*** TGAGCGGCTTACCTCTTAT	This work
spR	GTTTCTTC*CTGCAG*CGGCCGCTACTAGTCGCGTGGGCGGTATTCATCT	This work
prF	GTTTCTTC*CTGCAG*CGGCCGCTACTAGTAGCGTGCACCTGTTTTTATTTTCCTAATCTATGGTCC	This work
prR	GTTTCTTC**GAATTC**GCGGCCGCT ***TCTAGA*** GTGGCTTAAATCCTCCACCCC	This work
amF	GTTTCTTC**GAATTC**GCGGCCGCT ***TCTAGA*** CGTAATCCCACGCTGTTACA	This work
amR	GTTTCTTC*CTGCAG*CGGCCGCTACTAGTATTATTAAATCACCTCTTCGATAACCC	This work

Restriction enzymes recognition sites: *EcoRI* (Bold), *XbaI* (Bold, Italics), *SpeI* (Underlined)*,* and *PstI* (Italics).

Each genetic biopart was obtained by PCR and assembled by rounds of digestion with specific restriction enzymes (Fermentas) and ligations (T4 DNA ligase, Thermo Scientific). Briefly: 1) *malEp* was cut with *EcoRI* and *SpeI*, at the same time the vector pSB1A2, containing the RBS BBa_B0030 was cut with *EcoRI* and *XbaI*, and then ligated to obtain the “*malEp*-RBS” construction in the vector pSB1A2. 2) The same was done with *amyA* and the double terminator of the transcription BBa_B1002 to get the “*amyA*-double terminator” construction in the vector pSB1AK3. 3) The signal peptide *fhuDsp* was cut with *XbaI* and *PstI* and the *malEp*-RBS construction (in the vector pSB1A2 mentioned above) was cut with *SpeI* and *PstI*; these fragments were ligated to obtain the “*malEp*-RBS-*fhuDsp*” construction in the vector pSB1A2. 4) Finally, this last construction was cut with *SpeI* and *PstI*, and ligated with the digestion of *amyA*-double terminator to obtain the pAM plasmid. The final genetic construction encloses: the *malE* promoter, a RBS, the *fhuD* signal peptide, the alpha amylase gene *amyA* and the double terminator of the transcription in the backbone vector pSB1A2. Every construction was confirmed by PCR and restriction enzyme digestion analysis and the final construction by DNA sequencing.

### Amylase expression assays

SDS-PAGE was performed using 10% polyacrylamide in a Mini Protean Tetra Cell (BioRad) and stained with Coomassie blue R250. To determine the amylolytic activity of plates, starch-containing plates were stained with 1.5 mL of a 1:5 dilution of lugol solution (7.21, 5.17, and 86.6 g/L of I_2_, KI, and ethanol, respectively; Karal, México).

### Quantitative starch determination

Starch content was determined by its ability to complex iodine [33]. First, the maximum peak absorbance of soluble starch was determined by scanning a starch sample with lugol from 200 to 800 nm. Maximum absorbance was observed at 570 nm. Next, a calibration curve of starch was performed at 570 nm. To quantify starch, samples were centrifuged at 10,000 rpm for 5 minutes. Ten microliters of lugol (1:10 dilution) was added to the supernatant, and the absorbance was immediately measured at 570 nm (Beckman DU 640 Spectrophotometer).

### Glucose, maltose, and maltodextrin determination

Sugar content was determined by 3, 5-dinitrosalicylic acid (DNS) assays. Briefly, 0.5 mL of samples were mixed with 0.5 mL DNS reagent [10 g/L DNS (Sigma), 16 g/L NaOH (Karal, México), 300 g/L sodium potassium tartrate (Sigma)], boiled 5 min, and cooled on ice. Next, the mixture was diluted (1:5 dilution) and the absorbance at 540 nm was measured. The absorbance was compared with the calibration curve of each sugar. Maltodextrins in the culture media were analyzed using the HPLC system Agilent HP 1200 series (Agilent Technologies) equipped with Zorbax carbohydrate column (4.6 × 150 mm 5-micron PN 843300–908; Agilent Technologies) and a refraction index detector (RID). The mobile phase of 75:25 (v/v) acetonitrile: water was used and run using an isocratic gradient at a flow rate of 1.4 mL/min and 30°C.

## Competing interests

This work was partially financed by SIOSI S.A.P.I. de C.V.

## Authors’ contributions

LMRC designed the study, performed experiments, and drafted the manuscript. AMA conceived the study and helped prepare the manuscript. Both authors read and approved the final manuscript.

## References

[B1] FitzPatrick M Champagne P Cunningham MF Whitney RA A biorefinery processing perspective: treatment of lignocellulosic materials for the production of value-added products Bioresour Technol 2010 101 8915 8922 10.1016/j.biortech.2010.06.125 20667714

[B2] Rosales-Colunga LM Razo-Flores E Ordoñez LG Alatriste-Mondragón F De León-Rodríguez A Hydrogen production by *Escherichia coli* ΔhycA ΔlacI using cheese whey as substrate Int J Hydrogen Energ 2010 35 491 499

[B3] Rosales-Colunga L Alvarado-Cuevas Z Razo-Flores E León Rodríguez A Maximizing hydrogen production and substrate consumption by *Escherichia coli* WDHL in cheese whey fermentation Appl Biochem Biotechnol 2013 171 704 715 10.1007/s12010-013-0394-9 23881784

[B4] Federici F Fava F Kalogerakis N Mantzavinos D Valorisation of agro-industrial by-products, effluents and waste: concept, opportunities and the case of olive mill wastewaters J Chem Technol Biotechnol 2009 84 895 900 10.1002/jctb.2165

[B5] Ferreira-Leitão V Gottschalk L Ferrara M Nepomuceno A Molinari H Bon ES Biomass residues in Brazil: availability and potential uses Waste Biomass Valor 2010 1 65 76 10.1007/s12649-010-9008-8

[B6] Schievano A D’Imporzano G Adani F Substituting energy crops with organic wastes and agro-industrial residues for biogas production J Environ Manage 2009 90 2537 2541 10.1016/j.jenvman.2009.01.013 19254824

[B7] Orozco RL Redwood MD Leeke GA Bahari A Santos RCD Macaskie LE Hydrothermal hydrolysis of starch with CO2 and detoxification of the hydrolysates with activated carbon for bio-hydrogen fermentation Int J Hydrogen Energ 2012 37 6545 6553 10.1016/j.ijhydene.2012.01.047

[B8] Alavi S Starch research over the years Food Res Int 2003 36 307 308 10.1016/S0963-9969(02)00232-6

[B9] Mayer F Hillebrandt JO Potato pulp: microbiological characterization, physical modification, and application of this agricultural waste product Appl Microbiol Biotechnol 1997 48 435 440 10.1007/s002530051076 9390450

[B10] Tullman-Ercek D DeLisa MP Kawarasaki Y Iranpour P Ribnicky B Palmer T Georgiou G Export pathway selectivity of *Escherichia coli* twin arginine translocation signal peptides J Biol Chem 2007 282 8309 8316 10.1074/jbc.M610507200 17218314 PMC2730154

[B11] Boos W Shuman H Maltose/maltodextrin system of *Escherichia coli*: transport, metabolism, and regulation Microbiol Mol Biol Rev 1998 62 204 229 9529892 10.1128/mmbr.62.1.204-229.1998 PMC98911

[B12] Dippel R Bergmiller T Bohm A Boos W The maltodextrin system of *Escherichia coli*: glycogen-derived endogenous induction and osmoregulation J Bacteriol 2005 187 8332 8339 10.1128/JB.187.24.8332-8339.2005 16321937 PMC1316995

[B13] Dippel R Boos W The maltodextrin system of *Escherichia coli*: metabolism and transport J Bacteriol 2005 187 8322 8331 10.1128/JB.187.24.8322-8331.2005 16321936 PMC1316994

[B14] Wandersman C Schwartz M Ferenci T *Escherichia coli* mutants impaired in maltodextrin transport J Bacteriol 1979 140 1 13 387714 10.1128/jb.140.1.1-13.1979 PMC216772

[B15] Winkler J Kao K Computational identification of adaptive mutants using the VERT system J Biol Eng 2012 6 3 10.1186/1754-1611-6-3 22472487 PMC3351376

[B16] Wright BE Stress-directed adaptive mutations and evolution Mol Microbiol 2004 52 643 650 10.1111/j.1365-2958.2004.04012.x 15101972

[B17] Foster PL Adaptive mutation in *Escherichia coli* J Bacteriol 2004 186 4846 4852 10.1128/JB.186.15.4846-4852.2004 15262917 PMC451643

[B18] Lenski R Mongold J Sniegowski P Travisano M Vasi F Gerrish P Schmidt T Evolution of competitive fitness in experimental populations of *E. coli*: what makes one genotype a better competitor than another? Antonie Van Leeuwenhoek 1998 73 35 47 10.1023/A:1000675521611 9602277

[B19] Shibuya I Iimura Y Ishikawa T Ouchi K Matsuyama A Yamamoto T Morikawa M Nishiya T Isolation and characterization of starch-utilizing mutants of *Escherichia coli* Agric Biol Chem 1986 50 875 882 10.1271/bbb1961.50.875

[B20] Dardonville B Raibaud O Characterization of *malT* mutants that constitutively activate the maltose regulon of *Escherichia coli* J Bacteriol 1990 172 1846 1852 2180908 10.1128/jb.172.4.1846-1852.1990 PMC208678

[B21] Raha M Kawagishi I Müller V Kihara M Macnab RM *Escherichia coli* produces a cytoplasmic alpha-amylase, AmyA J Bacteriol 1992 174 6644 6652 1400215 10.1128/jb.174.20.6644-6652.1992 PMC207642

[B22] Nishino K Honda T Yamaguchi A Genome-wide analyses of *Escherichia coli* gene expression responsive to the BaeSR two-component regulatory system J Bacteriol 2005 187 1763 1772 10.1128/JB.187.5.1763-1772.2005 15716448 PMC1063996

[B23] Franchini AG Egli T Global gene expression in *Escherichia coli* K-12 during short-term and long-term adaptation to glucose-limited continuous culture conditions Microbiol 2006 152 2111 2127 10.1099/mic.0.28939-0 16804185

[B24] Vary P Biedendieck R Fuerch T Meinhardt F Rohde M Deckwer W-D Jahn D *Bacillus megaterium*—from simple soil bacterium to industrial protein production host App Microbiol Biotechnol 2007 76 957 967 10.1007/s00253-007-1089-3 17657486

[B25] Bachmann BJ Pedigrees of some mutant strains of *Escherichia coli* K-12 Microbiol Mol Biol Rev 1972 36 525 557 10.1128/br.36.4.525-557.1972 PMC408331 4568763

[B26] Baba T Ara T Hasegawa M Takai Y Okumura Y Baba M Datsenko KA Tomita M Wanner BL Mori H Construction of *Escherichia coli* K-12 in-frame, single-gene knockout mutants: the Keio collection Mol sys biol 2006 2 2006.0008 10.1038/msb4100050 PMC1681482 16738554

[B27] Registry of Standard Biological Parts http://parts.igem.org/Part:pSB1A2

[B28] Registry of Standard Biological Parts http://parts.igem.org/Part:pSB1AK3

[B29] Shetty RP Endy D Knight TF Jr Engineering BioBrick vectors from BioBrick parts J Biol Eng 2008 2 5 10.1186/1754-1611-2-5 18410688 PMC2373286

[B30] Phillips I Silver P A new BioBrick assembly strategy designed for facile protein engineering DSpace 2006 Cambridge, MA: MIT Artificial Intelligence Laboratory, MIT Synthetic Biology Working Group, Massachusetts Institute of Technology http://hdl.handle.net/1721.1/32535

[B31] Registry of Standard Biological Parts http://parts.igem.org/Part:BBa_B0030

[B32] Registry of Standard Biological Parts http://parts.igem.org/Part:BBa_B1002

[B33] Vukelić B Ritonja A Renko M Pokorny M Vitale L Extracellular α-amylase from *Streptomyces rimosus* App Microbiol Biotechnol 1992 37 202 204 10.1007/BF00178171 1368240

